# Sleepiness in Cognitively Unimpaired Older Adults Is Associated With CSF Biomarkers of Inflammation and Axonal Integrity

**DOI:** 10.3389/fnagi.2022.930315

**Published:** 2022-07-11

**Authors:** Diego Z. Carvalho, Erik K. St. Louis, Scott A. Przybelski, Timothy I. Morgenthaler, Mary M. Machulda, Bradley F. Boeve, Ronald C. Petersen, Clifford R. Jack, Jonathan Graff-Radford, Prashanthi Vemuri, Michelle M. Mielke

**Affiliations:** ^1^Department of Neurology, Mayo Clinic, Rochester, MN, United States; ^2^Center for Sleep Medicine, Division of Pulmonary and Critical Care, Department of Medicine, Mayo Clinic, Rochester, MN, United States; ^3^Department of Quantitative Health Sciences, Mayo Clinic, Rochester, MN, United States; ^4^Department of Psychology, Mayo Clinic, Rochester, MN, United States; ^5^Department of Radiology, Mayo Clinic, Rochester, MN, United States; ^6^Department of Epidemiology and Prevention, Wake Forest University School of Medicine, Winston-Salem, NC, United States

**Keywords:** sleepiness, inflammation, axonal integrity, interleukin-6 (IL-6), neurofilament light chain (NfL), Alzheimer’s disease, sleep disturbance

## Abstract

**Introduction:**

Sleepiness has been associated with cognitive decline and dementia in the elderly. Older adults with excessive daytime sleepiness appear to be more vulnerable to longitudinal amyloid PET accumulation before the onset of the dementia. However, it remains unclear whether sleepiness is similarly associated with other biomarkers of Alzheimer’s disease (AD), axonal integrity, and inflammation, which may also contribute to neurodegeneration and cognitive decline.

**Methods:**

In this cross-sectional analysis, we identified 260 cognitively unimpaired adults (>60 years) from the Mayo Clinic Study of Aging, a population-based cohort from Olmsted County (MN), who underwent CSF quantification of AD biomarkers (Aβ42, p-tau, p-tau/Aβ42) in addition to at least one of the following biomarkers [neurofilament light chain (NfL) interleukin-6 (IL-6), IL-10, and tumor necrosis factor-α (TNF-α)]. We fit linear regression models to assess associations between sleepiness, as measured by the Epworth Sleepiness Scale (ESS), and CSF biomarkers, controlling for age, sex, APOε4 status, body mass index, hypertension, dyslipidemia, and prior diagnosis of obstructive sleep apnea.

**Results:**

Higher ESS scores were associated with higher CSF IL-6 and NfL, but not with the other CSF biomarkers. For every ESS score point increase, there was a 0.009 ([95% CI 0.001–0.016], *p* = 0.033) increase in the log of IL-6 and 0.01 ([95% CI 0.002–0.018], *p* = 0.016) increase in the log of NfL. A sensitivity analysis showed an association between ESS scores and log of p-tau/Aβ42 only in participants with an abnormal ratio (>0.023), highly predictive of amyloid positivity. For every ESS score point increase, there was a 0.006 ([95% CI 0.001–0.012], *p* = 0.021) increase in the log of CSF p-tau/Aβ42.

**Conclusion:**

Sleepiness was associated with greater CSF IL-6 and NfL levels, which could contribute to neurodegeneration or alternatively cause sleepiness. Higher NfL levels may result from sleep disruption and/or contribute to sleepiness *via* disturbed connectivity or damage to wake-promoting centers. Associations between sleepiness and p-tau/Aβ42 in participants with abnormal ratio suggest that amyloid positivity contributes to vulnerability to sleep disturbance, which may further amyloid accumulation in a feed-forward loop process. Prospective studies of these markers are needed to determine cause-effect relationships between these associations.

## Introduction

A meta-analysis of 27 observational studies estimated that approximately 15% of Alzheimer’s disease (AD) in the population may be associated to sleep disturbance (Bubu et al., 2017). Sleepiness has long been recognized as a consequence of poor sleep quality in older adults (Pack et al., 2006), and has been associated with an increased risk for cognitive decline or dementia (Cohen-Zion et al., 2001; Foley et al., 2001; Ohayon and Vecchierini, 2002; Merlino et al., 2010; Elwood et al., 2011; Jaussent et al., 2012; Keage et al., 2012; Tsapanou et al., 2015). Additionally, wake-promoting neurons appear to be involved early in the AD process (Braak et al., 2011; Stratmann et al., 2016), and may be more vulnerable to AD pathology (Oh et al., 2019) and to prolonged exposure to repetitive hypoxemia (Veasey et al., 2004; Zhan et al., 2005; Zhu et al., 2007) or sleep disruption (Zhao et al., 2017), which may also contribute to daytime sleepiness.

We have previously shown that excessive daytime sleepiness (EDS) in older adults was associated with global cortical thinning, suggestive of accelerated brain aging (Carvalho et al., 2017). We also showed that EDS in the elderly was a predictor of longitudinal amyloid PET accumulation before the onset of dementia (Carvalho et al., 2018), in agreement with another study (Spira et al., 2018). These findings suggest that EDS in the elderly may be a clinical marker of increased vulnerability to neurodegeneration and AD pathology. We previously did not find an association between EDS and Tau-PET among cognitively unimpaired (CU) older adults (Carvalho et al., 2020), though we did not examine associations of EDS with CSF biomarkers of AD pathology (Aβ42, p-tau, p-tau/Aβ42), which may change earlier than neuroimaging biomarkers of *in vivo* pathology in pre-clinical stages of AD (Jack et al., 2013; Guo et al., 2021).

Pro-inflammatory cytokines, such as interleukin-6 (IL-6) and tumor necrosis factor-α (TNF-α), have been implicated in AD (Wang et al., 2015; Shen et al., 2019; de Oliveira et al., 2021) and neurodegeneration (Smith et al., 2012). Even anti-inflammatory cytokines, such as IL-10, may be abnormal in AD (Brosseron et al., 2014; Shen et al., 2019; Taipa et al., 2019). Neurofilament light chain (NfL) is released by injury to subcortical large-fiber axons (Hoffman et al., 1987), and has also emerged as a non-specific biomarker of neurodegeneration, white matter integrity and cognitive decline (Norgren et al., 2003; Mielke et al., 2019). It remains unclear, however, whether sleepiness in CU older adults may be associated with CSF biomarkers of AD pathology or extend to biomarkers of neuroinflammation or axonal integrity, which may also be associated with AD pathology, neurodegeneration, and/or cognitive changes.

The objective of this study was to identify associations between sleepiness in CU older adults with (1) CSF biomarkers of AD pathology (Aβ42, p-tau, p-tau/Aβ42); (2) CSF biomarkers of neuroinflammation (IL-6, IL-10, TNF-α); and (3) a CSF biomarker of axonal integrity and neurodegeneration (NfL).

## Materials and Methods

### Participant Selection

The Mayo Clinic Study of Aging (MCSA), which began in 2004, is a population-based cohort of residents living in Olmsted County (MN, United States). This study was approved by the Mayo Clinic and Olmsted Medical Center institutional review boards and informed consent was obtained from all participants or their surrogates. Details of the MCSA design have been published elsewhere (Roberts et al., 2008). For the present study, we included 260 CU participants older than 60 years of age without a diagnosis of neurological diseases who had available CSF inflammatory or NfL biomarkers, in addition to CSF Aβ42, p-tau, p-tau/Aβ42 (*n* = 251–260 depending on the biomarker) and completed the Epworth Sleepiness Scale (ESS) as part of the clinical assessment. There were no other exclusion criteria.

### Cognitive Status Determination

Cognitive status was determined by a consensus committee including the study coordinator, neuropsychologist, and the physician who evaluated each participant, as previously described (Roberts et al., 2008). Participants who performed within the normal range on a neuropsychological battery covering four domains (memory, language, executive function, and visuospatial) (Roberts et al., 2008) and, therefore, did not meet criteria for MCI (Petersen, 2004) or dementia (American Psychiatric Association, 1994) were deemed CU.

### Sleep Assessments

Participants completed the ESS (Johns, 1991) for assessment of subjective daytime sleepiness. They also responded to core questions of the Mayo Sleep Questionnaire (Boeve et al., 2013) assessing for general sleep disorder symptoms. Given that systematic objective assessment of obstructive sleep apnea (OSA) was not part of the original MCSA design, OSA diagnosis was obtained by an electronic health record algorithm based on ≥2 instances of specific International Classification of Diseases (ICD)-9 and/or ICD-10 diagnostic codes related to sleep apnea in separate dates in their electronic chart. The published algorithm has shown robust performance at identifying diagnosed cases at our site (positive predictive value = 100% [95% CI 97–100%]) (Keenan et al., 2020). From diagnosed cases, an apnea–hypopnea index (AHI) was collected, when available, for a sensitivity analysis. Limited availability of other objective sleep variables and treatment compliance data precluded systematic assessment of other parameters of OSA severity and management.

### APOε Status

APOε genotyping was performed as previously described (Crook et al., 1994). Participants with 1 or more APOε4 alleles were considered to have a positive APOε4 status.

### Medical Comorbidities Assessment

History of medical conditions was abstracted by trained nurses using the Rochester Epidemiology Project (REP) medical records-linkage system (St Sauver et al., 2012). Body mass index was obtained from measurements of height and weight by study coordinators at the clinical visit. Because of (1) the impact of cardiovascular health in cognitive outcomes and neurodegeneration in the aging population (Vemuri et al., 2015, 2017, 2019; Guzman-Martinez et al., 2019); and (2) the potential associations between sleep disturbance, cardiovascular health and inflammation (Grandner, 2017), we focused on medical comorbidities that affect cardiovascular health.

### CSF Assessment

CSF was obtained through lumbar punctures performed early in the morning in the lateral decubitus position. The sample was divided into 0.5-mL aliquots and stored at −80°C for future analyses avoiding freeze–thaw cycles prior to the current analyses. CSF Aβ42 and tau phosphorylated at threonine 181 (P-tau181) was measured with automated electrochemiluminescence Elecsys immunoassays (Roche Diagnostics) at Mayo Clinic Rochester. CSF IL-6, IL-10, and TNF-α levels were measured on the Simoa HD-1 platform (Quanterix, Lexington, MA, United States). The CSF NfL levels were measured in the Clinical Neurochemistry Laboratory at the University of Gothenburg using an in-house sandwich enzyme-linked immunosorbent assay (ELISA) (Gaetani et al., 2018). The level of p-tau-181 was divided by the level of Aβ42 to generate a p-tau/Aβ42 ratio biomarker, which has been found to be a better predictor of amyloid positivity than individual AD biomarkers when using an in-house cutoff (>0.023) (Campbell et al., 2021).

### Statistical Analysis

We performed cross-sectional analyses to examine associations between CSF biomarkers and sleepiness, as measured by ESS scores. Due to non-Gaussian or skewed distribution of CSF biomarkers, these variables were analyzed with logarithmic transformation, except for TNF-α, which could not be satisfactorily normalized and required non-parametric testing. Multivariable linear regression models were fit separately with each CSF biomarker (except TNF-α) as the dependent variable. ESS score was added as our independent variable of primary interest adjusting for age, sex, APOε4 genotype carrier, BMI, hypertension, dyslipidemia, and OSA diagnosis. As an initial step, we included all variables and then used backward elimination (probability of F set for variable entry at 0.05 and removal at 0.10) to obtain a final parsimonious model, less susceptible to over-fitting of data or unstable associations. Quantile regression was used to examine a possible association between TNF-α levels and ESS scores. For subgroup analyses of amyloid-positive participants or those with available AHI, unadjusted Pearson and Spearman’s rank correlations were initially performed to examine associations between two variables, according to data distribution. Subsequent analyses with either Pearson partial correlation or multiple variable linear regression were performed if initial unadjusted correlations were significant. Logarithmic transformation of variables were pursued, if necessary, to allow linear approximation. Owing to the exploratory nature of this work, adjustment for multiple comparisons were not performed. Statistical analyses were performed with SPSS software for Windows (version 28; IBM Corporation). A two-sided *p*-value < 0.05 was considered statistically significant.

## Results

The sample characteristics are summarized in Table 1. The participants’ mean (±SD) age was 73.3 (±6.8) years old. They were predominantly male (65%). A previous diagnosis of OSA was present in one quarter of the sample, which was consistent with the presence of symptoms of snorting or choking (23.6%) and witnessed apneas (17%).

**TABLE 1:** Demographic, clinical, and CSF biomarker characteristics.

Demographic characteristics	*N* = 260
Age, years, mean ± SD	73.3 ± 6.8
Sex, male, *n* (%)	169 (65)
APOε4, ≥1 allele, *n* (%)	71 (27.3)
Education, years, median (IQR)	15 (12–16)
BMI, kg/m^2^, mean ± SD	28.3 ± 4.7
**Medical comorbidities**	
Dyslipidemia, *n* (%)	210 (80.8)
Hypertension, *n* (%)	162 (62.3)
Diabetes, *n* (%)	38 (14.6)
OSA diagnosis, *n* (%)	65 (25)
AHI, hour^–1^, median (IQR)	19.5 (10–31.8)
**Sleep assessment**	
ESS scores, mean ± SD	5.9 ± 3.8
EDS, *n* (%)	47 (18.1)
**Mayo Clinic Sleep Questionnaire**	
Snorting or choking, *n* (%)	61 (23.6)
Witnessed apneas, *n* (%)	44 (17)
Restless legs, *n* (%)	16 (6.2)
Sleepwalking, *n* (%)	1 (0.4)
Dream enactment, *n* (%)	16 (6.2)
Nocturnal cramps, *n* (%)	85 (32.7)
**CSF biomarkers**	
Aβ42, pg/mL, median (IQR)	1096.5 (785.8–1549.0)
p-tau-181, pg/mL, median (IQR)	18 (14.4–23.2)
p-tau/Aβ42, median (IQR)	0.015 (0.012–0.022)
Amyloid positive, *n* (%)	60 (23.1)
IL-6, pg/mL, median (IQR)	2.4 (1.8–3.2)
IL-10, pg/mL, median (IQR)	0.22 (0.16–0.28)
TNF-α, pg/mL, median (IQR)	0.19 (0.19–0.25)
NfL, pg/mL, median (IQR)	532 (399.3–741.8)

*BMI, body mass index; AHI, apnea–hypopnea index (in subgroup of 54 participants with available data); ESS, Epworth Sleep Scale; EDS, excessive daytime sleepiness (ESS score ≥10); Aβ, amyloid beta; amyloid positive (p-tau/Aβ42 >0.023); IL, interleukin; TNF-α, tumor necrosis factor alpha; NfL, neurofilament light chain.*

**FIGURE 1:**
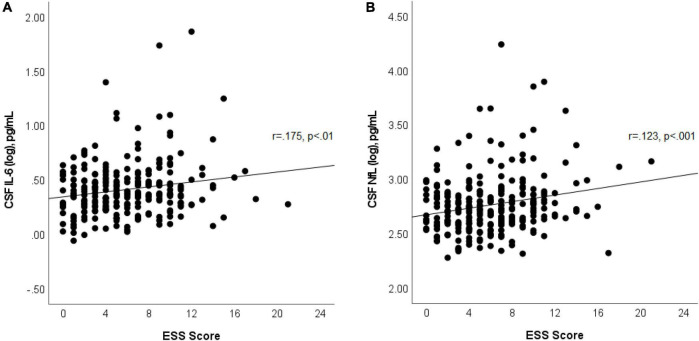
Scatterplot shows associations between ESS scores and log of CSF IL-6 **(A)** and log of NfL **(B)**. Best fit line is displayed, with the Pearson correlation coefficient and its *p*-value.

**FIGURE 2:**
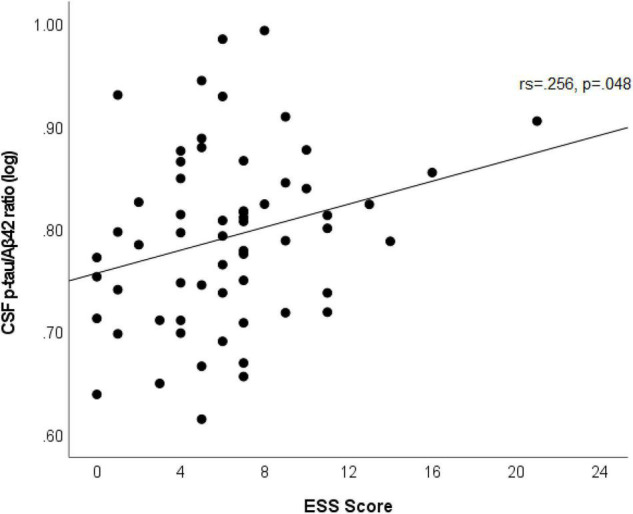
Scatterplot shows an association between ESS scores and log of CSF p-tau/Aβ42 in participants with abnormal ratio (amyloid positive). Best fit line is displayed, with the Spearman’s correlation coefficient and its *p*-value.

In multivariable linear regression analyses assessing for associations between CSF biomarkers (Aβ42, p-tau, p-tau/Aβ42, IL-6, IL-10, and NfL) and ESS scores, adjusted for age, sex, APOε4 genotype status, BMI, dyslipidemia, hypertension, and prior OSA diagnosis; we found significant associations between CSF IL-6 and NfL levels with ESS scores. These associations persisted after backward selection procedure (Table 2 and Figures 1A,B). For every ESS score increase, there was a 0.009 ([95% CI 0.001–0.016], *p* = 0.033) increase in the log of IL-6 and 0.01 ([95% CI 0.002–0.018], *p* = 0.016) increase in the log of NfL. There were no associations between ESS and any of the other biomarkers (β = −0.001 [95% CI −0.007 to 0.005], *p* = 0.674 for log of Aβ42; 0.002 [95% CI −0.005 to 0.009], *p* = 0.549 for log of p-tau; 0.002 [95% CI −0.005 to 0.008], *p* = 0.569 for log ofp-tau/Aβ42; and 0.003 [95% CI −0.003 to 0.009], *p* = 0.286 for log of IL-10). Given that amyloid status measured by amyloid PET strengthened the association between EDS and longitudinal amyloid PET changes in our previous study (Carvalho et al., 2018), we split the analyses examining the associations between ESS scores and CSF Aβ42, p-tau, and p-tau/Aβ42 based on amyloid status as measured by a CSF p-tau/Aβ42 > 0.023 cut-off, which had a 91% overall percent agreement with amyloid status from amyloid PET (Campbell et al., 2021). We first identified that ESS scores were significantly associated with log of p-tau/Aβ42 (Spearman’s rho = 0.256, *p* = 0.048) only in those with abnormal ratio (amyloid positive, *n* = 60) (Figure 2). In the multivariable analyses, ESS was not significantly associated with AD biomarkers in participants with a normal ratio (amyloid negative). However, in participants with an abnormal ratio (amyloid positive), ESS score was associated with CSF p-tau/Aβ42. For every ESS score increase, there was a 0.006 ([95% CI 0.001–0.012], *p* = 0.021) increase in the log of CSF p-tau/Aβ42. No associations were observed with other AD biomarkers.

**TABLE 2:** Estimates for final linear regression models after backward selection procedure.

	IL-6 (log)		NfL (log)	
Covariates	β (95% CI)	*p*-value	β (95% CI)	*p*-value
ESS scores	0.009 (0.001; 0.016)	0.033	0.010 (0.002; 0.018)	0.016
Age	0.008 (0.004; 0.013)	<0.001	0.011 (0.006; 0.016)	<0.001
Sex (male)	Not included		0.086 (0.020; 0.151)	0.011
APOε4 (any allele)	−0.062 (−0.130; 0.005)	0.069	Not included	

A diagnosis of OSA was not associated with any of the CSF biomarkers. In addition, in a sensitivity analysis replacing either snorting/choking or witnessed apneas for OSA diagnosis, the association between ESS scores with CSF IL-6 and NfL levels remained significant. We did not find a significant association between ESS scores and median CSF TNF-α using quantile regression in the multivariable model, controlling for the same variables included in the linear models (*b* = 0 [95% CI −0.001 to 0.002], *p* = 0.479).

In a subgroup analysis of 54 participants with available AHI from the original OSA diagnosis, ESS scores correlated with log of NfL after controlling for AHI (Pearson partial correlation *r* = 0.286, *p* = 0.038), but not with IL-6. There were significant correlations between AHI and ESS (Spearman’s rho = 0.31, *p* = 0.023), IL-6 (Spearman’s rho = 0.39, *p* = 0.004), NfL (Spearman’s rho = 0.33, *p* = 0.012), and Aβ42 (Spearman’s rho = 0.33, *p* = 0.016). However, these associations did not remain significant after we performed partial correlations adjusting for age and sex with AHI distribution normalized using common logarithmic transformation.

## Discussion

In this study, CU older individuals with sleepiness, as measured by the ESS scores, had higher levels of CSF IL-6 and NfL. In those participants with higher AD pathology as measured by abnormal p-tau/Aβ42, sleepiness correlated with higher ratios.

### Interleukin-6, Sleep, and Neurodegeneration

Growing evidence suggests that IL-6 is associated with sleepiness and poor sleep quality. Serum IL-6 has been shown to have a circadian pattern, with a morning trough (Nilsonne et al., 2016), potentially preceded by a peak at night (Straub and Cutolo, 2007). As such, experimental perturbations of sleep duration and slow-wave sleep have led to diurnal oversecretion of IL-6 (Vgontzas et al., 1999). Systemic illness with high IL-6 levels (Reincke et al., 1994) and exogenous administration of IL-6 (Spath-Schwalbe et al., 1998) have been associated with increased sleepiness and fatigue, respectively. Higher plasma IL-6 levels correlated with greater sleepiness, as measured by mean sleep latency during naps in narcolepsy, hypersomnia, and OSA patients (Vgontzas et al., 1997; Li et al., 2017). Patients with dementia who nap longer were found to have higher IL-6 levels (Li et al., 2017). Thus, IL-6 could contribute to poor sleep quality by its effects on sleep architecture, characterized by a delay or reduction in slow wave sleep, and a delay or reduction in REM sleep with an overall decrease in sleep efficiency (Spath-Schwalbe et al., 1998; Hong et al., 2005; von Kanel et al., 2006). Interestingly, Tocilizumab, a humanized anti-IL-6 receptor (anti-IL-6R) monoclonal antibody, reduces sleepiness in patients with rheumatoid arthritis (Townes et al., 2012), which may be related to improvements in sleep quality due to better control of sleep disturbance caused by pain and discomfort.

A meta-analysis of 72 studies (*n* > 50,000) showed that sleep disturbance was associated with increased serum/plasma IL-6 levels (Irwin et al., 2016). OSA (Vgontzas et al., 1997; Liu et al., 2000; Li et al., 2017; Motamedi et al., 2018), insomnia (Vgontzas et al., 2002), and narcolepsy (Okun et al., 2004) have also been associated with increased serum IL-6 levels. In OSA, IL-6 has been associated with hypoxemia and obesity (Vgontzas et al., 1997). Although a causal relationship cannot be established in most studies due to their cross-sectional nature, a study showed a reduction of IL-6 levels with CPAP therapy (Yokoe et al., 2003). Unfortunately, other studies investigating associations between sleep disturbance and CSF IL-6 (not serum) are non-existent. Because serum IL-6 is a sensitive marker of acute and chronic systemic inflammation (Gabay, 2006; Zhu et al., 2009), it may or may not adequately reflect intrathecal IL-6 production, depending on how significant neuroinflammation in the central nervous system is and how it compares to systemic inflammation. When compared to blood samples, CSF IL-6 levels have been found to be significantly higher in patients with multiple sclerosis (Stelmasiak et al., 2000), subarachnoid hemorrhage (Hopkins et al., 2012), ventriculostomy-related infection (Hopkins et al., 2012), bacterial meningitis (Beran et al., 2009), traumatic brain injury (Kossmann et al., 1995), and stroke (Tarkowski et al., 1995). In stroke patients, CSF IL-6 predicted stroke size while serum IL-6 did not (Tarkowski et al., 1995). Overall, the literature suggests that CSF IL-6 level is more sensitive and specific to intrathecal IL-6 production than serum/plasma levels.

In the central nervous system, IL-6 is produced primarily by the microglia and astrocytes in response to damage signals (including Aβ oligomers) (Toro et al., 2001; Vukic et al., 2009) to promote repair and homeostasis. IL-6 has been shown to contribute to the production of Aβ (Ringheim et al., 1998; Ait-Ghezala et al., 2007), hyperphosphorylation of tau (Quintanilla et al., 2004), vascular pathology (Su et al., 2021), and upregulation of neuroinflammation, with overactivation of protein kinases and increased oxidative stress which can contribute to neurodegeneration (Akiyama et al., 2000; Spooren et al., 2011; Erta et al., 2012; Wang et al., 2015; Guzman-Martinez et al., 2019). Although (1) AD patients have been found to have increased serum IL-6 levels in a meta-analysis of 175 studies (Lai et al., 2017), and (2) serum IL-6 has been associated with an increased risk of dementia (Engelhart et al., 2004) and worse cognitive function in AD patients (Lai et al., 2017), it was not associated with longitudinal cognitive decline or increased MCI risk in our CU population (Wennberg et al., 2019). CSF IL-6 associations with amyloid status and AD dementia have also been inconsistent (Martinez et al., 2000; Galimberti et al., 2008; Wennstrom et al., 2015; Janelidze et al., 2018). In our study, CSF IL-6 did not correlate with CSF AD biomarkers (Spearman’s rho = 0.053, *p* = 0.4 for Aβ42; Spearman’s rho = −0.014, *p* = 0.829 for p-tau; and Spearman’s rho = −0.05, *p* = 0.427 for Aβ42/p-tau). It appears that the relationship between IL-6, AD and neurodegeneration depend on other pro-inflammatory and anti-inflammatory cytokines and different endophenotypic expression of inflammatory markers (Wang et al., 2015; Peng et al., 2020), in addition to IL-6 receptor polymorphisms (Su et al., 2016; Haddick et al., 2017; Zhang et al., 2021).

### Neurofilament Light Chain, Sleep, and Neurodegeneration

Neurofilament light chain may be associated with sleep disturbance, but the literature has shown contradictory findings. Middle-aged insomnia patients have higher NfL levels compared to those without insomnia (Zhang et al., 2018; Ren et al., 2022), and one study reported decreased NfL levels after treatment (Zhang et al., 2018). In another study, higher NfL levels were associated with worse subjective sleep quality, and decreased sleep duration, sleep efficiency, and REM sleep (Zhang et al., 2018). In patients with multiple sclerosis, higher serum NfL levels were also associated with lower sleep efficiency in addition to lower NREM sleep contribution (Sacmaci et al., 2020). However, another study in chronic insomnia did not show associations with subjective sleep quality (Ren et al., 2022). Serum NfL levels have been found to positively correlate with the apnea, hypopnea and 4%-oxygen desaturation indices in addition to the percent of total sleep time with oxyhemoglobin saturation below 90%, but these correlations were mostly driven by moderate-to-severe OSA patients (Arslan et al., 2021). This may explain why we did not find associations between CSF NfL and a diagnosis of OSA, without a measure of severity, in our multivariable linear regression models. In the same study, NfL did not correlate with sleep efficiency or ESS scores (Arslan et al., 2021), but might have lacked power to detect associations, because analyses had to be split by sleep apnea severity. In another study, CSF NfL levels did not differ between patients with versus without narcolepsy type 1 (Baiardi et al., 2020), who are known to have significant sleep fragmentation with poor sleep efficiency. Plasma NfL levels in 4712 middle-aged and elderly non-demented persons were not associated with subjective sleep quality, actigraphy-estimated sleep and 24-h activity rhythms after adjusting for multiple confounders (Lysen et al., 2020). Nevertheless, compared to self-rated normal time in bed (7–9 h), spending a long time in bed (>9 h) was associated with higher NfL levels (Lysen et al., 2020). A longer time in bed may indicate an increased need for sleep, a potential manifestation of more severe sleepiness, or also suggest more sedentary behavior or an overall decreased functional status.

It remains unclear how sleep disturbance may contribute to CSF NfL. The fact that insomnia is associated with elevated levels of CSF NfL suggest that it may be cleared by the glymphatic system like amyloid (Xie et al., 2013; Albargothy et al., 2018) and tau (Iliff et al., 2014). The relationship between NfL and parameters of hypoxemia in OSA patients suggest a different mechanism, potentially *via* increased oxidative stress and neuroinflammation causing neurodegeneration (Daulatzai, 2015; Snyder et al., 2017) and white matter disease (Rostampour et al., 2020; Zacharias et al., 2021), which may contribute to sleepiness (Xiong et al., 2017). Although tau-related neurodegeneration of wake-promoting neurons can start in pre-clinical stages of AD (Braak et al., 2011; Stratmann et al., 2016), and could contribute to sleepiness (Oh et al., 2022), it is unknown whether it could be associated with higher NfL levels. We have shown that CU late middle-aged and older adults with EDS have decreased cortical thickness (Carvalho et al., 2017). Given that CSF NfL has been associated with longitudinal cortical thinning in pre-demented elderly (Mielke et al., 2019), greater neurodegeneration or accelerated aging may link sleepiness to higher CSF NfL. NfL levels have also been associated with longitudinal changes in cognition, AD pathology (Jin et al., 2019; Mielke et al., 2019) and white matter integrity (Zetterberg et al., 2016; Mielke et al., 2021), as well as with an increased risk for MCI (Kern et al., 2019; Lim et al., 2021) and dementia (de Wolf et al., 2020; Lim et al., 2021). Our finding of elevated CSF NfL levels among participants who reported greater sleepiness is in agreement with previous literature indicating that EDS is a risk factor for cognitive decline and dementia (Cohen-Zion et al., 2001; Foley et al., 2001; Ohayon and Vecchierini, 2002; Merlino et al., 2010; Elwood et al., 2011; Jaussent et al., 2012; Keage et al., 2012; Tsapanou et al., 2015).

Although the studies discussed above used either serum/plasma NfL or CSF NfL, we found a moderate correlation between plasma and CSF NfL (Spearman’s rho = 0.568, *p* < 0.001) in 79 elderly participants without dementia (median age 76.5 [IQR 71.7–80.7]) (Mielke et al., 2019). However, it remains unclear whether CSF levels could be more sensitive to detect subtle changes related to sleep. Moreover, it has been suggested that NfL may be subjected to different levels of dilution when released into the systemic circulation, which is related to BMI (Manouchehrinia et al., 2020). Levels may also be partially affected by renal function in older adults (Akamine et al., 2020). We cannot exclude the possibility that these potential confounders related to serum NfL might have contributed to some of the inconsistent findings from the literature.

### Sleepiness and p-tau/Aβ42

Although sleepiness was not associated with either CSF Aβ42 or p-tau levels, it was associated with p-tau/Aβ42 in patients with an abnormal ratio. This abnormal ratio has been found to be a better predictor of amyloid positivity than individual AD biomarkers (Campbell et al., 2021). This subgroup analysis shows that amyloid status is important in this association, which is in agreement with our previous study showing that EDS was associated with longitudinal amyloid PET signal change, after controlling for baseline amyloid status (Carvalho et al., 2018), and not with tau PET signal (Carvalho et al., 2020). Sleepiness may be associated with increases in amyloid due to an increase in synaptic activity (Kamenetz et al., 2003; Cirrito et al., 2005; Kang et al., 2009; Bero et al., 2011), decrease in slow-wave sleep (Varga et al., 2016), impairment of the glymphatic system (Xie et al., 2013), altered CSF dynamics or due to hypoxemia (Shiota et al., 2013; Osorio et al., 2014; Liguori et al., 2017). It is also possible that individuals with higher amyloid levels are more susceptible to the effects of sleep disturbance, leading to more sleepiness and additional AD pathology in a feed-forward loop mechanism, and/or that higher baseline brain amyloid load predisposes to greater subsequent sleepiness and sleep disturbances, including OSA. This feed-forward loop hypothesis is corroborated by another study that showed that severity of OSA was associated with greater brain amyloid PET deposition only in amyloid positive participants (Sharma et al., 2018). Additionally, participants with higher amyloid levels are more likely to have increased tau pathology affecting wake-promoting neurons (Stratmann et al., 2016; Oh et al., 2019, 2022), which could also contribute to sleepiness.

### Limitations

Limitations of the study warrant consideration. First, the cross-sectional design of this study does not allow us to determine whether the associations found are causal. For instance, we cannot determine if sleepiness is a marker of more severe sleep disturbance that leads to an increase in neuroinflammation, axonal damage, AD pathology, and neurodegeneration; or if an abnormal neuroinflammatory response, connectivity issue caused by axonal injury, and/or neurodegeneration of wake-promoting centers results in more sleep disturbance and/or daytime sleepiness. Larger prospective longitudinal cohort studies will be necessary to determine the directionality of these associations. Second, we relied on self-reported sleepiness as opposed to objective measures of sleepiness [e.g., multiple sleep latency test (MSLT)], which might have obscured some associations. Older individuals may underestimate their sleepiness as measured by ESS. In a cross-sectional observational study including 104 independently living non-demented older subjects with daytime sleepiness complaints and 104 non-demented close relatives (CRs), subjects rated their sleepiness with ESS scores significantly lower (7.10 ± 4.31) than their CR proxy did (9.70 ± 5.14) (Onen et al., 2013). However, in-laboratory objective measures of sleepiness such as the MSLT have been criticized for their suboptimal performance (Bonnet, 2006; Trotti et al., 2013) and may not be a good surrogate of sleepiness in real life scenarios (Johns, 1994). Third, although we used a validated electronic health record algorithm that had high positive predictive value to identify patients with a diagnosis of OSA (Keenan et al., 2020), the frequency of OSA in our cohort (25%) is probably underestimated due to underdiagnosis. We also lacked data concerning severity of hypoxemia, which could have further impacted upon or perhaps mediated the associations. Fourth, detailed information about CPAP prescription, compliance and response to treatment was not available, which might have limited associations between OSA diagnosis and biomarkers. Finally, the lack of adjustment for multiple comparisons might have increased the probability of a type I error. Our study, however, has significant strengths and novelty. Most studies examining associations between the biomarkers under study, in particular IL-6 and NfL, and sleep disturbance have small sample sizes and insufficient control of potential confounders, generating concern for spurious associations and publication bias. Further, few studies have examined the association between sleep and CSF inflammatory or neurodegeneration biomarkers.

## Conclusion

Our study suggests a complex relationship between sleepiness and CSF IL-6 and NfL. Higher IL-6 and NfL may be associated with sleepiness either as a cause or consequence of underlying sleep disturbance. Both may be associated with hypoxemia, which should be further assessed in future studies. It is also possible that higher NfL suggests greater underlying axonal injury and neurodegeneration that contributes to daytime sleepiness by disrupting attention networks or output from wake-promoting regions. Although there is evidence that both IL-6 and NfL contribute to neurodegeneration, the relationship with AD pathology is more consistent for NfL than IL-6, likely given IL-6 complex interactions with other inflammatory markers and receptor polymorphisms. Our results corroborate previous findings indicating that elderly individuals with daytime sleepiness are more likely to have greater AD pathology, and raise the possibility for increased vulnerability to neuroinflammation, neurodegeneration, and axonal injury.

## Data Availability Statement

Raw and analyzed de-identified data from the Mayo Clinic Study on Aging can be requested using the following link: https://ras-rdrs.mayo.edu/Request/IndexRequest. The request will be reviewed by the Mayo Clinic Study on Aging investigators and Mayo Clinic to verify whether the request is subject to any intellectual property or confidentiality obligations. A data sharing agreement must be obtained prior to release.

## Ethics Statement

The studies involving human participants were reviewed and approved by the Mayo Clinic and Olmsted Medical Center Institutional Review Boards. The participants provided their written informed consent to participate in this study.

## Author Contributions

DC, PV, and MiM: study concept and design. All authors: data collection or interpretation and critical revision of the manuscript for important intellectual content. DC: drafting of the manuscript. DC and SP: statistical analysis. All authors agreed to be accountable for the content of the work.

## Conflict of Interest

ES has received research support from Mayo Clinic CCaTS, NIH, the Michael J. Fox Foundation, and Sunovion, Inc. MaM receives research funding from the NIH. BB has served as an investigator for clinical trials sponsored by Alector and Biogen. He serves on the Scientific Advisory Board of the Tau Consortium. He receives research support from the NIH the Mangurian Foundation, the Little Family Foundation, and the Ted Turner and Family Foundation. RP consults for Roche, Inc., Merck, Inc., Genentech, Inc., and Biogen, Inc., GE Healthcare and receives royalties from Oxford University Press for the publication of Mild Cognitive Impairment. CJ consults for Lily and serves on an independent data monitoring board for Roche but he receives no personal compensation from any commercial entity. JG-R receives research funding from NIH and serves on the editorial board of Neurology. PV receives research funding from NIH (NIA and NINDS). MiM served as a consultant to Biogen, Brain Protection Company, and LabCorp and receives research support from the NIH and DOD. The remaining authors declare that the research was conducted in the absence of any commercial or financial relationships that could be construed as a potential conflict of interest.

## Publisher’s Note

All claims expressed in this article are solely those of the authors and do not necessarily represent those of their affiliated organizations, or those of the publisher, the editors and the reviewers. Any product that may be evaluated in this article, or claim that may be made by its manufacturer, is not guaranteed or endorsed by the publisher.
